# CRISPR/Cas9 nickase mediated targeting of urokinase receptor gene inhibits neuroblastoma cell proliferation

**DOI:** 10.18632/oncotarget.25647

**Published:** 2018-06-29

**Authors:** Karina D. Rysenkova, Ekaterina V. Semina, Maxim N. Karagyaur, Anna A. Shmakova, Daniyar T. Dyikanov, Petr A. Vasiluev, Yury P. Rubtsov, Kseniya A. Rubina, Vsevolod A. Tkachuk

**Affiliations:** ^1^ Lomonosov Moscow State University, Faculty of Medicine, Laboratory of Gene and Cell Technologies, 119991, Moscow, Russian Federation; ^2^ Federal State Budgetary Organization National Cardiology Research Center Ministry of Health of the Russian Federation, Institute of Experimental Cardiology, 121552, Moscow, Russian Federation; ^3^ Institute of Regenerative Medicine, Lomonosov Moscow State University, 119991, Moscow, Russian Federation; ^4^ Shemyakin-Ovchinnikov Institute of Bioorganic Chemistry of the Russian Academy of Sciences, 117997, Moscow, Russian Federation

**Keywords:** CRISPR/Cas9, genome editing, urokinase receptor, cell proliferation, neuroblastoma

## Abstract

Neuroblastoma is a tumor arising from pluripotent sympathoadrenal precursor cells of neural cell origin. Neuroblastoma is one of the most aggressive childhood tumors with highly invasive and metastatic potential. The increased expression of urokinase and its receptor is often associated with a negative prognosis in neuroblastoma patients.

We have shown that targeting of the *Plaur* gene in mouse neuroblastoma Neuro 2A cells by CRISPR/Cas9n results in ~60% decrease in cell proliferation (p<0.05), reduction in the number of Ki-67 positive cells, caspase 3 activation and PARP-1 cleavage. Knockout of uPAR leads to downregulation of mRNA encoding full-length TrkC receptor, which is involved in p38^MAPK^ and Akt signalling pathways. This finding provides a rationale to study a role of uPAR in neuroblastoma progression, since uPAR could be considered a potential therapeutic target in neuroblastoma treatment.

## INTRODUCTION

Neuroblastoma is the most common pediatric extracranial solid tumour originating from the sympatoadrenal lineage residing in neural crest [1–3]. It accounts for 7–15% of childhood cancers [2, 4]. Neuroblastoma is prone to spontaneous regression or differentiation into a benign ganglioneuroma, when diagnosed in infancy. However, in older patients neuroblastoma can be metastatic with rapid progression and fatal outcome. Pharmacological agents, such as retinoic acid, NO, phenylacetate, can also induce neuroblastoma cell differentiation or apoptosis in culture as well as tumor regression in clinics [5–8]. Currently, even the most intensive multimodal therapy results in only modest improvement in the cure rate of aggressive neuroblastoma [9].

The mechanisms responsible for the aggressive neuroblastoma behavior are poorly understood. Statistically, significant association between low patient survival rate and MYCN oncogene amplification, diploid DNA content, allelic loss, undifferentiated cell histology and changes in Trk receptors’ profiles have been reported [9]. A family of neurotrophic growth factors and their receptors, playing an important role in neural development, has been implicated in pathogenesis and progression of neuroblastoma [10–14]. This family consists of four members: nerve growth factor (NGF), brain-derived neurotrophic factor (BDNF), Neurotrophin-3 (NT3) and Neurotrophin-4/5 (NT4/5). Neurotrophin effects are mediated by binding to two classes of neurotrophin receptors (NTRs) – p75NTR and Trks (tyrosine kinase receptors), the latter comprising TrkA, TrkB and TrkC [15].

Several studies indicate that tumor cells utilize proteolytic enzymes for the degradation of extracellular matrix and invasion. Elevated levels of urokinase (uPA) and urokinase receptor (uPAR) have been reported in basalioma, melanoma, glioblastoma, different types of carcinomas, prostate, lung, ovarian, breast and gastrointestinal cancers [16–34]. uPA and uPAR were overexpressed in highly invasive metastatic forms of human neuroblastoma [3]. Increased expression of uPA or its receptor has been associated with elevated metastatic potential and poor prognosis in these patients. Binding of uPA to uPAR accelerates activation and increases the enzymatic activity of uPA. Experimental approaches using small interfering RNA to inhibit uPAR expression or blocking antibody to impair uPAR function can significantly interfere with glioma/glioblastoma invasion *in vivo* and *in vitro* and can also downregulate intracellular signalling leading to reduced tumor vascularization, suppress cell survival and proliferation [16, 19, 28, 29, 35]. These and other data indicate that the uPAR intervention aimed at reduction of its expression in cancer cells may represent potentially promising new approach to anticancer therapy. Although siRNA approach is effective in uPAR suppression, it has some drawbacks, since reduction in gene expression is not stable and siRNA effect drops down rapidly in actively proliferating cells.

A significant advance in genome engineering was made upon development of CRISPR/Cas9 system for nuclease-based genome editing and transcriptional regulation [36, 37]. The RNA-guided CRISPR/Cas9 (clustered regularly interspaced short palindrome repeats) technology provides an effective means for introduction of targeted loss-of function mutations into the genes of interest. These mutations, and hence, biological effects are heritable, highly specific and ensure complete gene shut-off in contrast to partial reduction of gene expression by other methods [38]. The CRISPR/Cas9 nickase (Cas9n introduces single strand breaks to DNA) genome editing system combines two plasmids each harbouring Cas9n gene and chimeric guide RNA (sgRNA). These sgRNAs are complementary to DNA sequences next to obligate PAM (protospacer adjuscent motif) trinucleotides. CRISPR-Cas9n makes two single-strand breaks with minimal off-target effects within the target DNA, followed by activation of non-homologous end joining (NHEJ) repair system. NHEJ inserts or removes a few nucleotides to Cas9n cleavage sites leading to a farameshift mutations and premature termination of translation [36, 39–43]. This approach can be used effectively for high precision loss-of-function genetic studies in cell lines and primary cultures, in animal disease models, for whole-genome mutation screening in cancer cell and genome editing *in vivo* [37, 39, 42, 44–46].

Recent advances using CRISPR/Cas9 system have opened new perspectives from basic research to clinical application. Inactivation of EPH1 with CRISPR/Cas9 technology suppressed ovarian cancer cell proliferation, invasion and migration *in vitro* [46]. In breast cancer cells, CRISPR/Cas9 system has been applied to disrupt HER2 oncogene expression. Ablation of HER2 resulted in inhibition of MAPK/Erk and PI3K/Akt signalling cascade, reduced cell proliferation and decreased tumorigenicity [45]. CRISPR/Cas9 technology has been used for genetic correction of a dominant mutation in *Crygc* gene that causes cataract in mice [37]. The first human trial using CRISPR/Cas9 gene editing to treat metastatic non-small-cell-lung cancer has been launched in China in 2016 [47]. In the current study we employed CRISPR/Cas9n system to target *Plaur* gene in Neuro 2A neuroblastoma cells. We created plasmids for uPAR gene inactivation, selected genetically modified clones and tested the efficiency of uPAR targeting using CRISPR/Cas9n. We showed that CRISPR/Cas9n targeting of *Plaur* gene resulted in inhibition of neuroblastoma proliferation, significant reduction in the number of Ki-67 positive cells, caspase 3 activation and PARP-1 cleavage. uPAR downregulation correlated with the decrease in TrkC mRNA level and Akt phosphorylation.

## RESULTS

### Targeting of *Plaur* by CRISPR/Cas9n and selection of modified clones

**Figure 1:**
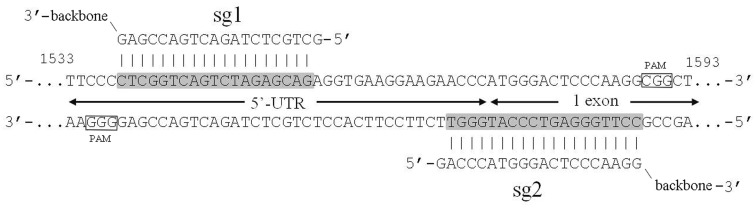
gRNAs and targeted region of *Plaur* Designed gRNAs are complimentary to the first exon (sg2) and 5’-UTR (sg1) of the *Plaur*. Boundary of 5’UTR and exon1 of Plaur is shown by arrows. Binding sites of sgRNAs in genomic DNA are shadowed in grey. The start codon is highlighted, PAM sequences are framed.

In the current study we designed pX458nickase-sg1 and pX458nickase-sg2 constructs to selectively target *Plaur* and disrupt uPAR function in Neuro 2A cells. These constructs also drove expression of EGFP, which was used as a selection marker to sort out cells transfected with components of CRISPR/Cas9n genome editing tool. CRISPR/Cas9n application was predicted to result in a frameshift mutation close to the start codon of *Plaur* and to cause premature termination of uPAR translation. Specific DNA regions recognized by sg1 and sg2 were separated by 13 nucleotides, which were sufficient to induce double-strand breaks in the *Plaur* and to activate the NHEJ repair (Figure 1). The analysis of on-target sites and most probable off-target sites of *Plaur* sgRNAs are presented in Supplementary Figure 1 and Supplementary Figure 2, respectively.

**Figure 2:**
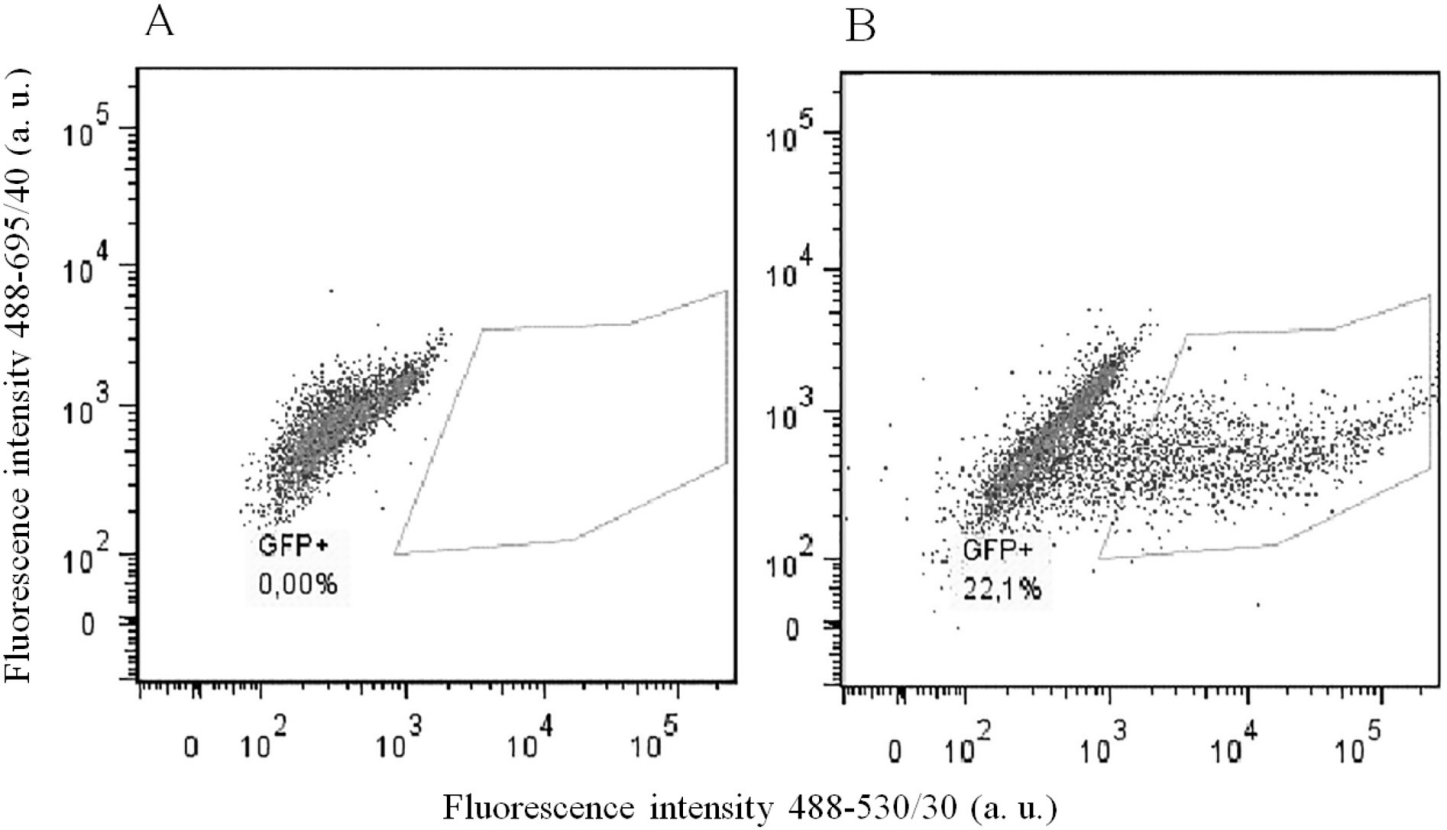
Flow cytometry analysis and sorting of EGFP-positive Neuro 2A cells **(A)** wild type Neuro 2A cells. **(B)** Neuro 2A cells co-transfected with sg1 and sg2 plasmids. Sorting gate for EGFP-positive cells is shown. Fluorescence emission in 530/30 channel is shown on the x-axis, and in 695/40 channel on the y-axis; excitation - 488 nm laser.

**Figure 3:**
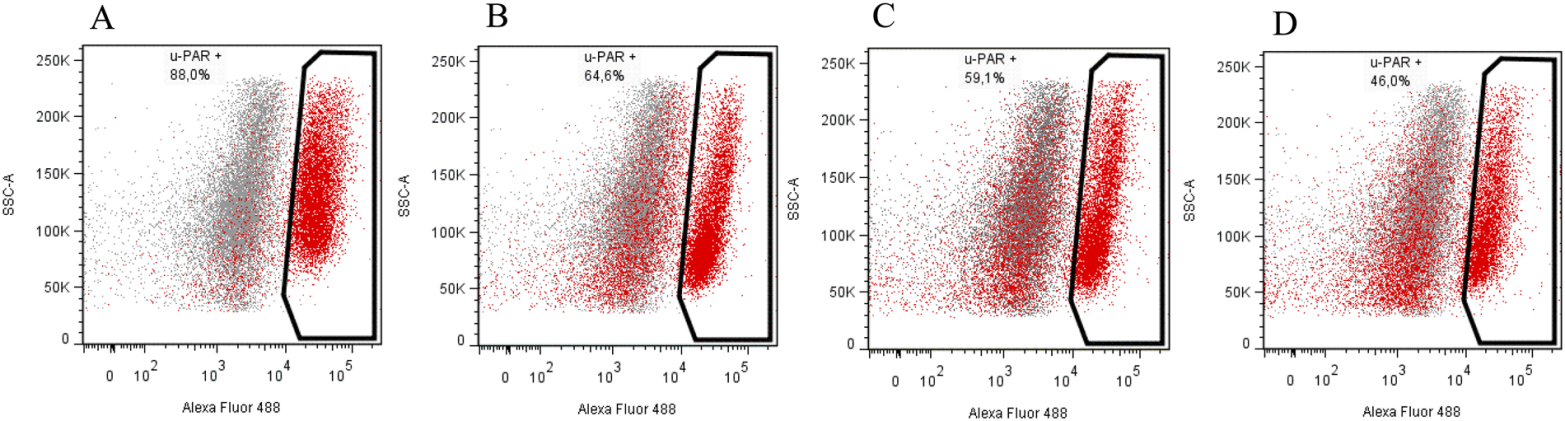
Subsequent co-transfection with sgRNA and Cas9n constructs reduces uPAR expression on the surface of Neuro 2A cells **(A)** wild type Neuro 2A cells. **(B, C, D)** s1, s2 and s3 cell populations, correspondingly. Fluorescence emission in 530/30 channel is shown on the x-axis and side-scatter is shown on the y-axis; excitation – 488nm laser.

To prove that the designed constructs effectively induced mutations in *Plaur*, we used them for Neuro 2A cell co-transfection. EGFP-positive cell with diminished uPAR cell surface expression were selected using FACS sorting. Neuro 2A is a line of immortalized tumor cells, characterized by an unstable karyotype of 94-98 chromosomes in the stemline, and 59-193 chromosomes in individual subclones (ATCC^®^ CCL-131™, Manassas, Virginia). The copy number of loci containing *Plaur* gene was expected to vary from one to several. Therefore, we carried out three sequential co-transfections with pX458nickase-sg1 and pX458nickase-sg2 to maximize targeting of multiple *Plaur* copies. uPAR expression was assessed using immunofluorescent staining with anti-uPAR antibody of EGFP-expressing cells after each round of co-transfection. Sorting gates and results of anti-uPAR staining are presented in Figures 2 and 3. Wt, s1, s2 and s3 correspond to Neuro 2A cell subpopulations of wild type (Figure 3A), cells after the first (Figure 3B), the second (Figure 3C) and the third co-transfection (Figure 3D). The proportion of uPAR-positive cells in control culture was 88%, while after the first, the second and the third round of transfection it decreased to 64.6%, 59.1%, and 46.0%, respectively (Figure 3). The efficacy of uPAR suppression was low and not sufficient to study phenotypic and functional changes. To sort out this problem we generated uPAR-deficient single-cell clones.

**Figure 4:**
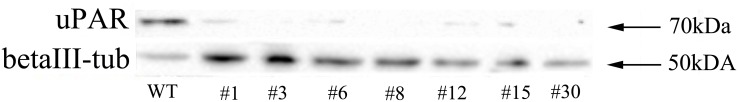
Analysis of uPAR expression in selected Neuro 2A clones by Western blot followed by detection with anti-uPAR antibodies Control Neuro 2A cells; 1, 3, 6, 8, 12, 15, 30 – Neuro 2A clones after co-transfection and single cell clone expansion. Selected clones showed reduced uPAR expression from moderate decrease to complete uPAR loss. β3-tubulin was used as loading control. Typical results from three independent experiments are presented.

To obtain uPAR-deficient clones, single-cell dilution of EGFP-positive s3 cells into 96-well plates at 1 cell per well was performed. After single cell clone expansion, 30 clones were selected for further analysis. uPAR protein expression level was detected by Western blotting. While some clones showed only moderate decrease in uPAR expression (data not shown), others demonstartated convincing decrease or complete loss of uPAR (clones 1, 3 6, 8, 12, 15, 30) (Figure 4). uPAR mRNA expression in three chosen uPAR-deficient clones (3, 6 and 30) ranged from a significant decrease to a complete loss as confirmed by RT-PCR analysis (Supplementary Figure 3).

Sanger's sequencing analysis of Plaur sgRNAs on-target sites using ChromasLite and TIDE programs (https://tide.deskgen.com/) revealed the uPAR knockout in clone #6 and 75% suppression of uPAR in clone #30 (Supplementary Figure 1). Precisely, the three cycles of gene modification using CRISPR/Cas9 nickase plasmid resulted in the 62-nucleotide deletion in *Plaur* gene in clone #6. The detection of a single peak in clone #6 in chromatograms (Supplementary Figure 1) corresponds to the only allele variant. In clone #30, three allele variants have been identified: two alleles bearing identical inserts of 4 nucleotides; one allele with 6 nucleotide deletion potentially restoring the reading frame; and one allele with 19 nucleotide deletion.

To predict the most probable *Plaur* sgRNAs off-target sites COSMID software (https://crispr.bme.gatech.edu/) was implemented. No mutations in the loci of the most likely off-target activity of the CRISPR/Cas9n system targeted by the chosen sgRNAs could be detected (Supplementary Figure 2).

Thus, the strategy of pX458nickase-sg1 and pX458nickase-sg2 transfection, EGFP-based cell sorting and clonal selection was effective and allowed us to obtain cells with varying uPAR expression levels.

### Downregulation of uPAR inhibits proliferation of Neuro 2A cells

**Figure 5:**
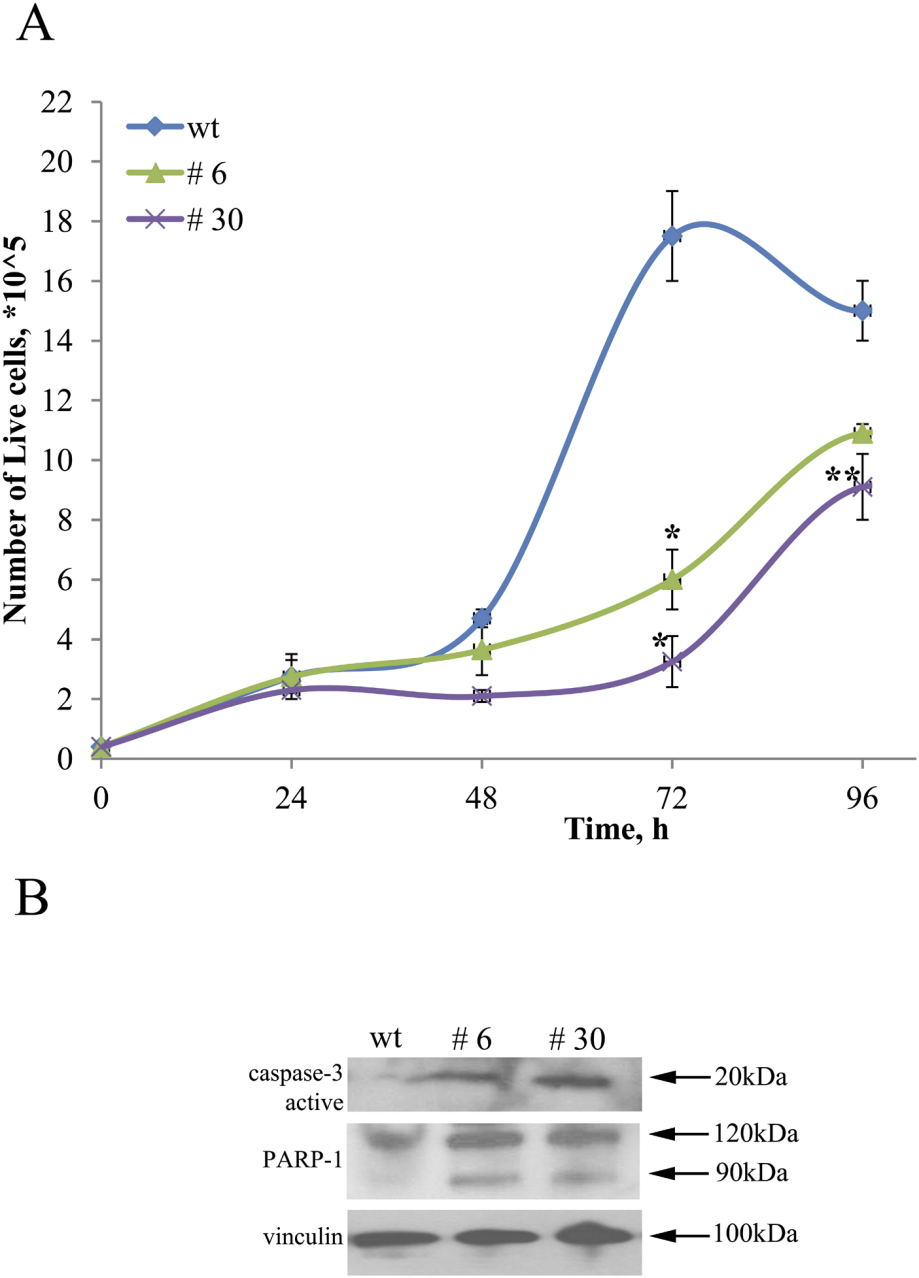
uPAR knockout restrains proliferation of Neuro 2A cells **(A)** ControlNeuro 2A cells; #3, #30 – uPAR-deficient clones. Reproducible results of three independent experiments are presented. The data are presented as Mean ± SEM (n=3 wells per group). ^*^ - p<0.001 versus control, ^**^- p<0.05 versus control (Newman-Keuls test). **(B)** Western blot demonstrating caspase-3 activation, PARP-1 cleavage and accumulation of 89 kDa fragment in uPAR-deficient clones compared to wt cells. Vinculin was used as loading control. Typical result from three independent experiments is presented.

**Figure 6:**
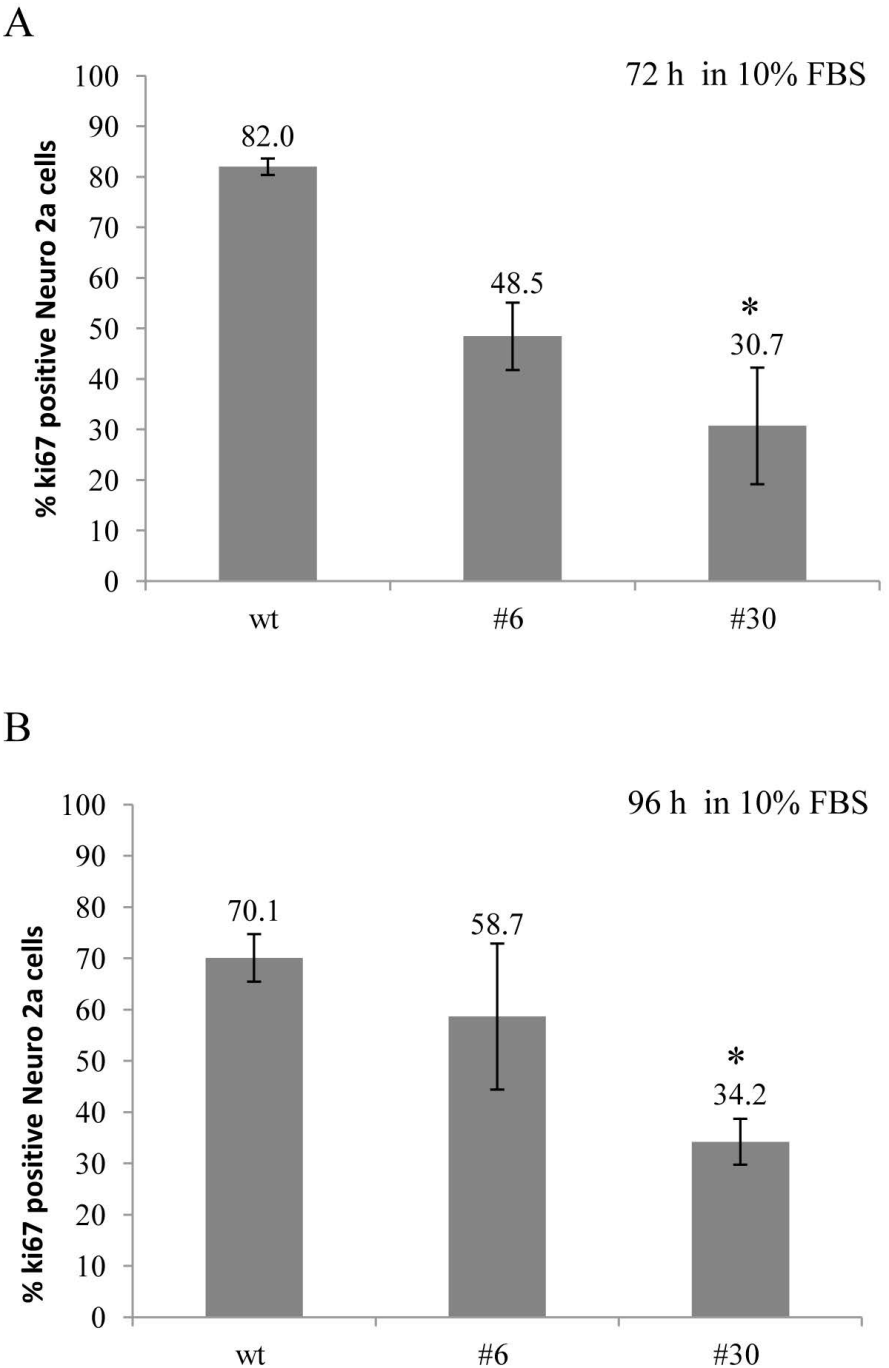
uPAR knockout results in a decreased amount of Ki-67-positive Neuro 2A cells Ki-67 expression was assessed in uPAR-deficient clones (#6, #30) and in wt cells in standard culture media (10% FBS) after 72 and 96 hours. Cells were fixed, stained with antibody against Ki-67 and subjected to FACS analisys. **(A)** The bar chart depicts the percentage of Ki-67-positive Neuro 2A cells in each cell type after 72 hours; **(B)** The percentage of Ki-67-positive Neuro 2A cells in each cell type after 96 hours. The data are presented as Mean ± SD (n=4 wells per group), ^*^p < 0.05 by ANOVA test.

**Figure 7:**
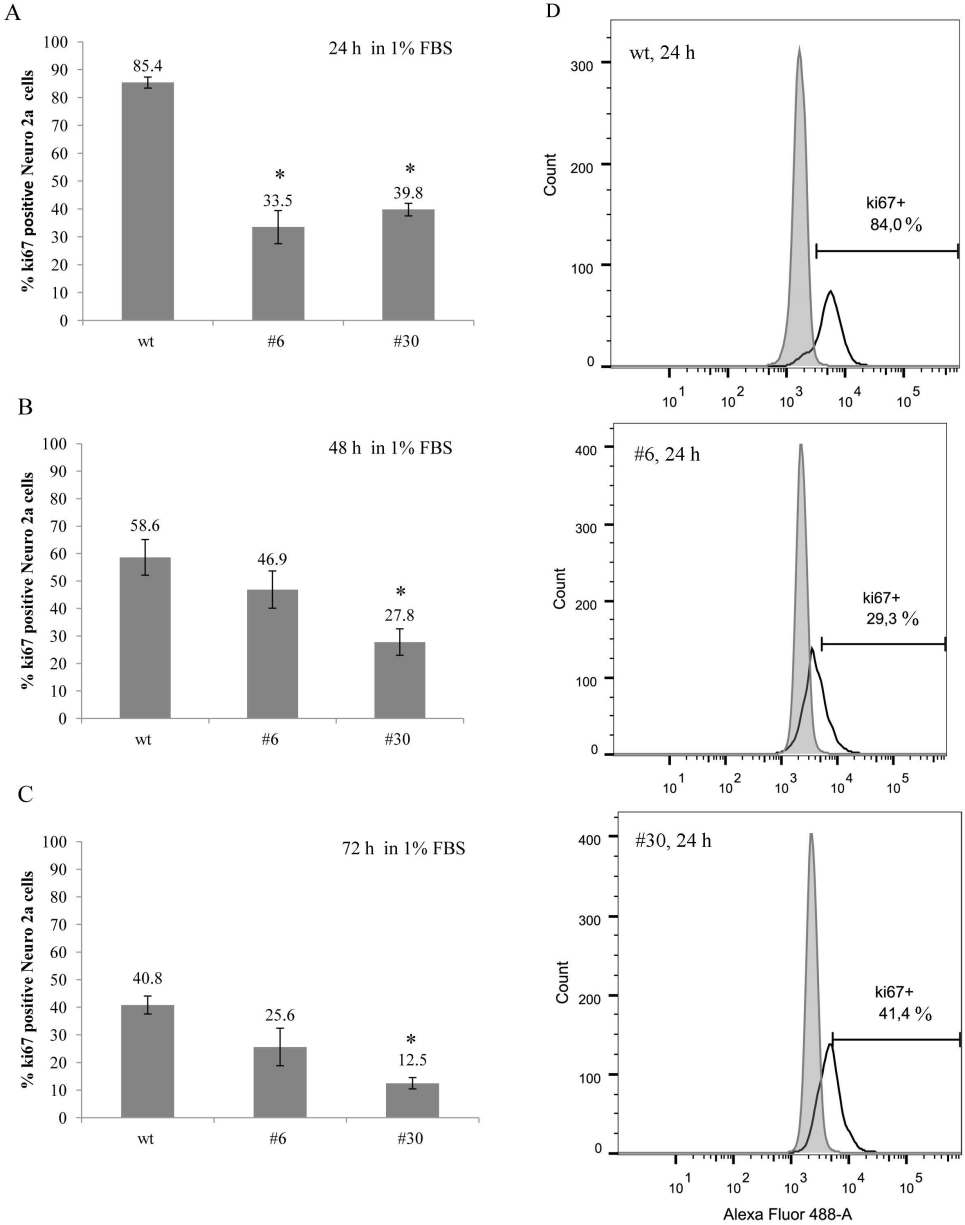
uPAR knockout results in a decreased amount of Ki-67-positive cells (low serum conditions) **(A-C)** The bar charts depict Ki-67 expression level in uPAR-deficient clones (#6, #30) and in wt cells after 24, 48 and 72 hours in low serum conditions (1% FBS). The data are presented as Mean ± SD (n=3 wells per group), ^*^ p < 0.05 ANOVA test. **(D)** Histograms depict the percentage of Ki-67-positive Neuro 2A cells in each cell type after 24 hours. Fluorescence emission in 530/30 channel is shown on the x-axis, and the cell count is shown on the y-axis; excitation - 488 nm laser. Typical results from three independent experiments are presented.

Literature data indicate that uPAR expression is elevated in high-risk neuroblastomas and is associated with enhanced invasive/metastatic potential and overall negative patient's prognosis [3]. To evaluate the pathophysiological significance of uPAR knockout on Neuro 2A cells, we compared the proliferation rate of uPAR-deficient clones (#6 and #30) to control Neuro 2A cells (wt) (Figure 5A, Figure 6, Figure 7). Decline in proliferation rate is obvious from the data showing live cell counts at different time points (equal number of cells were plated in control and experimental cultures) (Figure 5A). After 72 hours up to 5.3 times difference in live cell numbers was evident between control Neuro2A and clone #30. Similar results on the decreased cell counts were obtained for s2 and s3 subpopulations and clone #22 after 96 hours in culture. We observed an approximate 23% reduction in the number of cells for s2 and s3 subpopulations, compared to s1 and control cells; 33% decrease for clone #22 compared to the control (data not shown).

To further confirm these results, we used antibody against Ki-67 – a classic marker of cell proliferation that has been widely applied in cancer diagnostic and research [48]. We examined the proportion of uPAR-deficient and control cells, which entered the cell cycle after 72 and 96 hours. Ki-67 expression was determined using direct immunostaining followed by FACS analysis (Figure 6). After 72 hours the level of Ki-67 expression was lower in uPAR-deficient clones #6 and #30 (48.5±6.7% and 30.7±11.5%, correspondingly) compared to 82±1.6% in wt cells (p < 0.05, ANOVA) (Figure 6A). The decline in Ki-67 expression in clones #6 and #30 was also detected after 96 hours: 58.7±14.2% and 34.2±4.5% compared to 70.1±4.6 % Ki-67 level in wt cells (Figure 6B).

Due to the known effect of serum starvation on cancer cells *in vitro* resulting in their enhanced sensitization to standard chemotherapeutic agents [49], we performed the same experiment with Ki-67 cell staining and FACS analysis using low serum culture conditions (1% FBS). In low serum the difference in Ki-67 expression between uPAR-deficient clones and wt cells became even more pronounced (Figure 7). After 24 hours in culture, Ki-67 expression level in uPAR-deficient clones #6 and #30 was 33.5±5.9% and 39.8±2.3% Ki-67, respectively; while in wt cells Ki-67 was expressed in 85.4±2.0% of cells (p < 0.05, ANOVA) (Figure 7A). Further analysis revealed the gradual decline of Ki-67 expression in wt cells: from 85.4% after 24 hours to 40.8% after 72 hours in culture. Similarly, Ki-67 expression declined in uPAR-deficient clones from approximarely 30% (24 hours) to 12.5% (72 hours) (Figure 7B, 7C).

The difference in the amount of live cells in culture could be attributed not only to the decrease in cell proliferation rate but also to the induction of apoptosis. Actual execution of apoptosis depends upon activation of effector caspases, particularly caspase 3 [50]. Caspase 3 is primarily responsible for the cleavage of Poly (ADPribose) polymerase (PARP) during programmed cell death [51]. To test if uPAR knockout has any effect on the induction of apoptosis, we analysed the expression level of caspase 3 and the extent of PARP-1 cleavage in protein extracts using Western blot. We detected a significant increase of caspase 3 in uPAR-deficient clones. PARP-1 cleavage by caspase 3 (89 kDa fragment accumulation) was significantly higher in #6 and #30 clones compared to the control cells (Figure 5B).

### uPAR-deficient Neuro 2A cells exhibit decreased expression of TrkC

**Figure 8:**
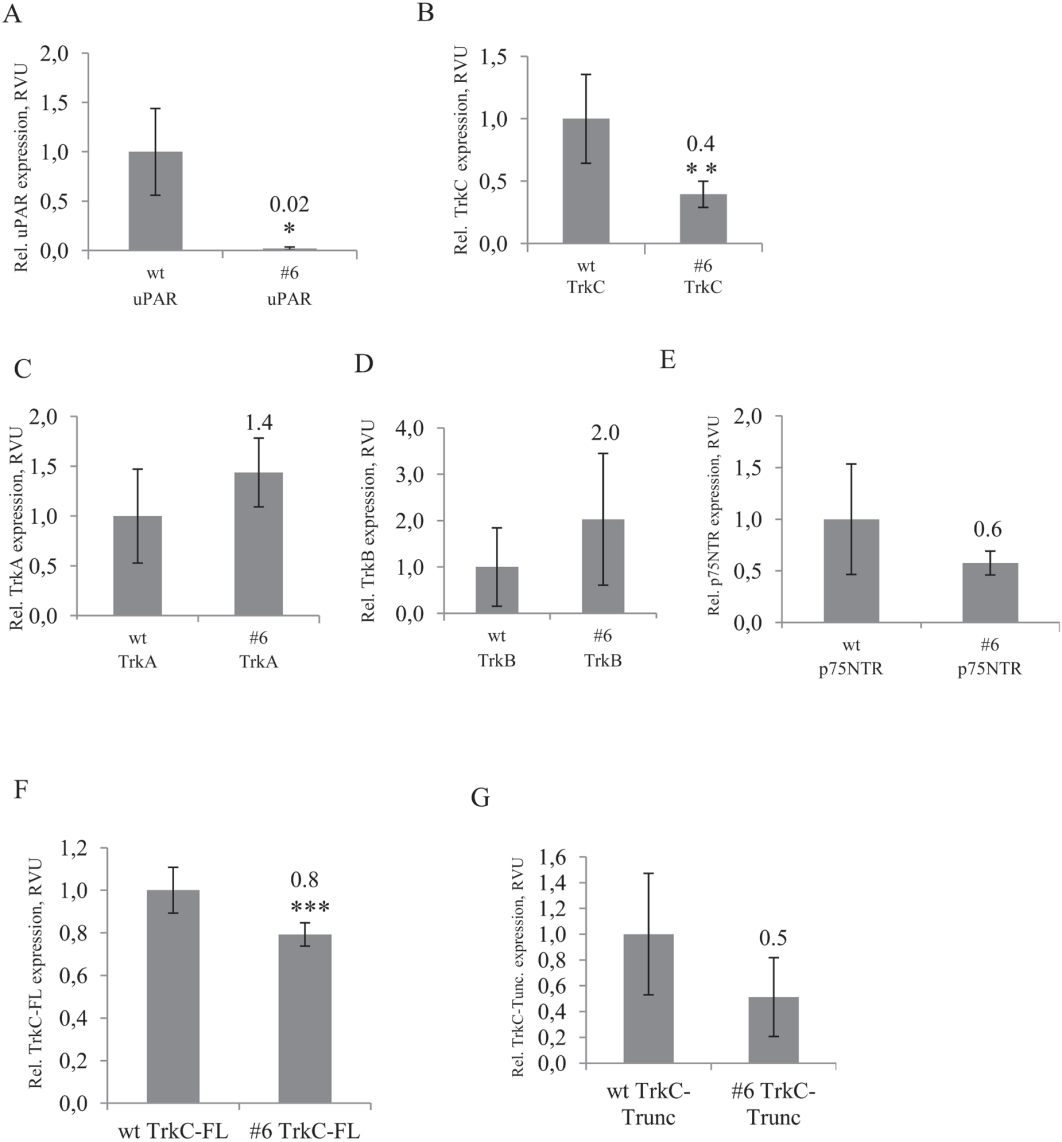
RT-PCR analysis of Trks mRNA expression shows significunt reduction of full length TrkC mRNA in uPAR-deficient cells **(A)** uPAR expression in control Neuro2A and uPAR-deficient clone #6, **(B)** TrkC expression in control cells and #6 clone, **(C)** TrkA expression in control (wt) and #6 clone, **(D)** TrkB expression in control (wt) and #6 clone, **(E)** p75NTR expression in control (wt) and #6 clone, **(F)** TrkC-FL (full-length TrkC) expression in control (wt) and #6 clone, **(G)** TrkC-Trunc expression (truncated form of TrkC) in control (wt) and #6 clone. The data are presented as Mean ± SD (n=3 wells per group), ^*^ p < 0.006; ^**^ p < 0.02; ^***^ p < 0.05 (Student's t-test). The mRNA level was normalized to the expression of the housekeeping gene β-actin.

Trk family kinases are known to be responsible for the regulation of neuroblastoma survival/apoptosis, proliferation and differentiation [11–14, 52, 53]. To identify the potential mechanisms responsible for reduced proliferation of uPAR-deficient Neuro 2A cells, we examined the mRNA expression level of Trks extracted from control cells and uPAR-deficient clones (#3, #6, #30). We also analysed the mRNA expression level of Nanog, the main pluripotency factor in Neuro 2A cells [54]. It appeared that uPAR knockout was accompanied by a 2.5-fold decrease in TrkC mRNA (Figure 8, Supplementary Figure 4), while no statistically significant difference in mRNA expression of TrkA, TrkB and p75NTR could be detected in uPAR-deficient cells compared to the control (Figure 8C, 8D, 8E). In addition, Nanog mRNA remained unchanged (data not shown). Given that the full-length and the truncated form of TrkC may be differently expressed in favourable and aggressive neuroblastomas [12, 52], we used primers designed to differentiate between the splice variants of the full-length TrkC (TrkC-FL) and truncated TrkC form (TrkC-Trunc) that lack tyrosine kinase domain (Figure 8F, 8G). No statistically significant difference could be detected in mRNA expression of TrkC-Trunc, while there was a 1.25-fold reduction in the functional TrkC-FL mRNA in uPAR-deficient clones.

**Figure 9:**
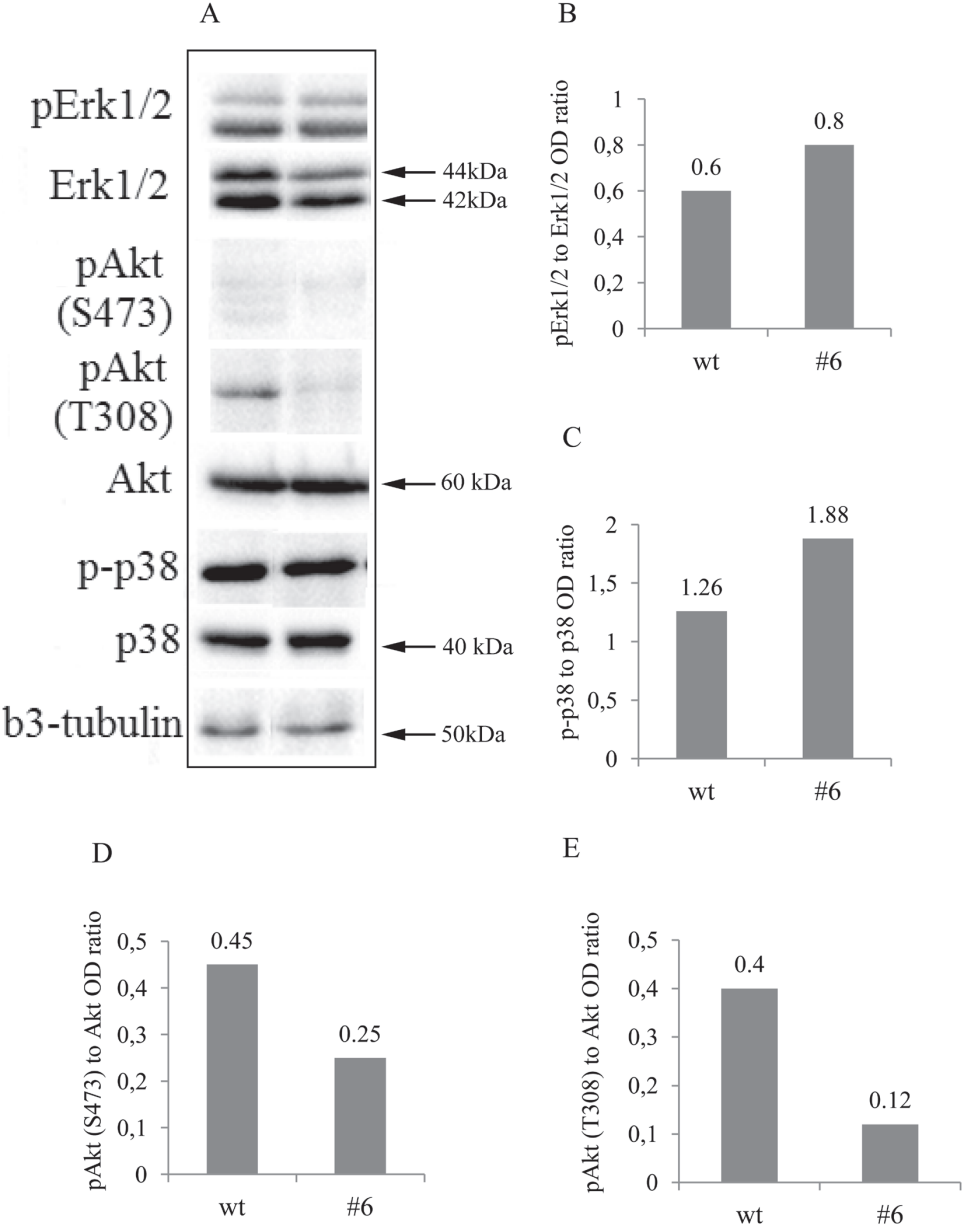
Western blot and densitometry analysis of Erk1/2, Akt, p38MAPK and their phosphorylated forms showed decreased activation of Akt and increased of p38MAPK **(A)** Western blots showed a marked reduction of Akt phosphorylation on both, Thr308 and Ser473 residues and a slight increase in phospho-p38^MAPK^ in uPAR-deficient clone #6 compared to the contol. Erk1/2 was not significuntly altered. β3-tubulin was used as loading control. Typical results from three independent experiments are presented. **(B-E)** densitometry analysis of the blots further confirming the obtained results.

According to the classic neurotrophic view, TrkC interaction with its NT-3 ligand may result in activation of the PI3K/Akt, MAPK/Erk1/2 and p38^MAPK^ signalling pathways in neuroblastoma cells [11, 52]. To identify critical signalling pathways involved in decreased neuroblastoma cell proliferation, we measured phosphorylation of Akt, Erk1/2 and p38^MAPK^ using Western blot analysis. The representative results are shown in Figure 7A. There was significant decrease in p-Akt in uPAR deficient clones compared to the control: Ser473 1.8- and Thr308 3.3-fold decline in phosphorylation (Figure 9A, 9D, 9E). Erk1/2 phosphrylation level didn't change significantly (Figure 9A and 9B), while there was approximately a 1.5-fold increase in p38^MAPK^ phosphorylation in uPAR-deficient clones compared to the control (Figure 9A and 9C).

## DISCUSSION

Several reports on uPAR mRNA and uPAR protein expression, on kinetic analysis of uPA-uPAR binding, and on the implementation of RNAi and uPAR antisense-expressing plasmids to inhibit uPAR expression, point to the important role of uPAR in the progression of malignant tumors of the nervous system such as astrocytoma, neuroblastoma, glioma and glioblastoma [3, 16, 55, 56]. In the present study we used CRISPR/Cas9 technology to suppress uPAR in neuroblastoma cells in order to gain a better understanding of the mechanisms responsible for neuroblastoma proliferation. The designed gRNAs were proved to selectively target *Plaur* in Neuro 2A cells. Analysis of the sequence data revealed uPAR complete knockout in clone #6 and 75% suppression in clone #30 (Supplementary Figure 1). We also analyzed the possible CRISPR/Cas9 off-target sites. No mutations in the loci of the most likely off-target activity of the CRISPR/Cas9n system targeted by the chosen sgRNAs could be detected (Supplementary Figure 2).

Targeting of the *Plaur* results in significant reduction in proliferative activity of Neuro 2A cells with the maximum effect in uPAR-deficient clones (Figures 5, 6, and 7). Proliferation rate was evaluated using live cell counts and Ki-67 immunostaining followed by FACS. Ki-67 antigen is specifically expressed in proliferating cells in G1, S, G2 and M phase of the cell cycle, whereas it is absent from non-cycling cells in G0 [48]. Both methods demonstrated the decline in the amount of live cycling cells. We also addressed a question if uPAR knockout can induce apoptotic cell death. First, we showed accumulation of active caspase 3 in uPAR-deficient clones compared to the control. Caspase mediated apoptosis is accomplished through the cleavage of several key proteins required for cell survival and functioning. PARP-1 is known to be involved in preventing DNA degradation and resisting to apoptosis. Cleavage of PARP-1 by caspases is known to be a hallmark of apoptosis [51, 67]. Accumulation of 89 kDa fragment of PARP-1 was significantly elevated in #6 and #30 clones compared to the control cells (Figure 5B). These results indicate that uPAR knockout has an impact on neuroblastoma cell survival and proliferation, which is in accordance with the previously published results on reduced tumor growth and invasion upon uPAR downregulation in glioma, glioblastoma and other cancers [16, 22, 27, 35, 57]. However, adenoviral vectors and RNAi-based strategies used in these studies have serious disadvantages, such as transient expression and inability to integrate into the genome [58]. The CRISPR/Cas9 technology used in the current study lacks these shortcomings [44, 59] and could be a promising tool for genetic engineering and cancer studies *in vitro* and *in vivo*.

Trk receptors and their ligands are known to be involved in neuroblastoma proliferation, survival/apoptosis and differentiation [2]. High TrkA and TrkC expression levels are associated with favourable clinical and biological features of neuroblastoma [52, 60]. Tumors with high level of TrkA are often subjected to spontaneous regression. TrkA-expressing tumor cells in the presence of NGF undergo neuronal differentiation, while NGF deprivation leads to apoptosis [2, 14, 53]. Co-expression of p75NTR and TrkA in neuroblastoma cells is associated with good prognosis. Overexpression of p75NTR alone leads to apoptosis in line with p75NTR structural homology to the TNF-R/Fas family of death receptors [2, 61]. TrkB and BDNF co-expression is detected in more aggressive neuroblastomas especially with MYCN amplification [2, 14]. As opposed to TrkA and TrkB, TrkC is considered to be a dependence receptor: in the presence of NT3 a positive differentiation or survival signal is transduced, while in the absence of the ligand TrkC induces apoptosis [11]. In the earlier studies, using Northern blot analysis low levels of TrkA and TrkC in Neuro 2A cells were detected [62]; the expression of p75NTR in these cells was verified using immunostaining [63]. Later it has been reported that Neuro 2A cells express p75NTR, TrkB and TrkC, but no TrkA [14]. Our data indicate that mRNA of all Trk receptors (TrkA, TrkB, TrkC, p75NTR) can be detected in Neuro 2A cells. All neurotrophins (NGF, BDNF, NT3 NT4/5) are present in Neuro 2A cells and can stimulate cell survival and proliferation [14]. The reduced proliferation of uPAR-deficient Neuro 2A cells in our experimental settings correlates with a decrease in mRNA TrkC content (Figure 8B), probably resulting from a lack of survival signalling via TrkC.

Truncated forms of Trks have an important biological role in neuroblastoma behaviour. Expression of truncated TrkB, that is generated by alternative splicing and serves as a decoy for BDNF, correlates with a more favourable outcome in neuroblastoma patients [64]. In contrast to tumor-suppressing role of full-length TrkA, a constitutively-active TrkAIII splice form in primary neuroblastomas and in SH-SY5Y cell line, antagonizes NGF/TrkA signalling and promotes tumor progression [13]. A subset of unfavourable neuroblastomas expresses a truncated form of TrkC, lacking tyrosine kinase domain [52, 60]. The main function of truncated TrkC is the inhibition of full-length TrkC signalling, achieved via a ligand-sequestering or a dominant-negative mechanism. Given that the full-length TrkC is increased while truncated TrkC is decreased in human neuroblastomas upon exposure to retinoic acid that limits proliferation and induces differentiation [12], we compared mRNA expression of full-length TrkC and truncated TrkC in control and in uPAR-deficient clones (Figure 8). We used primers designed to differentiate between the splice variants of the full-length TrkC and truncated TrkC, described earlier [65]. Statistically significant difference between control and uPAR-deficient cells was detected only for full-length TrkC, but not for truncated TrkC (Figure 8F and 8G), thus further supporting the assumption of the uPAR-mediated TrkC signaling that tranduces pro-survival and proliferating stimuli in Neuro 2A cells.

The cytoplasmic part of Trk receptors recruits signaling complexes, which activate PI3K/Akt, MAPK/Erk1/2 and p38MAPK pathways [11, 14, 52, 66]. Akt pathway is considered to be a clinically relevant and promising target for neuroblastoma treatment [4, 67, 68]. Akt is a serine/threonine kinase that is often hyperactivated in aggressive neuroblastomas, primary and metastatic tumors and cell lines [4, 67–69]. Phosphorylation of Akt at Thr308 (catalytical domain) is necessary and sufficient for Akt activation, whereas phosphorylation at Ser473 (C-terminal regulatory domain) is not sufficient but is required for optimal Akt activation [70]. Akt Thr308 up-regulates mammalian target of rapamycin (mTORC1) and p70S6K, which enhance protein synthesis. Phosphorylation of Akt at Ser473 by mTORC2 promotes anti-apoptotic and cell survival pathways [4, 68, 69]. *In vitro* activation of PI3K/Akt pathway is associated with enhanced survival and proliferation of SH-SY5Y, SK-N-BE(2) and Neuro 2A neuroblastoma cell lines [69, 71], while *in vivo* PI3K/Akt inhibition reduces tumor growth and MYCN protein expression [72]. In the current study, the decrease in cell proliferation in uPAR deficient clones was accompanied by a significant (3.3-fold) reduction of Akt phosphorylation at Thr308 residue and a less pronounced (1.8-fold) decrease in Akt phosphorylation at Ser473 (Figure 9A and 9D); the total Akt content remained unchanged. The obtained data suggest that the uPAR-dependent reduction in Neuro 2A cell proliferation can be responsible for the decrease in Akt phosphorylation.

Activating mutations in Ras-MAPK signalling pathways have been predicted in relapsing neuroblastomas resistant to chemotherapy [73]. Proliferative activity of neuroblastoma NB69 cell line can be inhibited by application of p38^MAPK^ pharmacological inhibitors [74]. A combination of p38^MAPK^ inhibitors with etoposide, a standard chemotherapy agent, strongly increases the sensitivity of neuroblastoma to chemotherapy and allows lowering the drug dose and overall toxicity [75]. Apoptotic pathway, activated in neuroblastoma SH-SY5Y cell line upon exposure to H_2_O_2_ can be blocked by cell pretreatment with p38^MAPK^ pharmacological inhibitors, indicating p38^MAPK^ involvement in regulation of cell death/survival response [76]. In our experiments there was approximately a 1.5-fold increase in p38^MAPK^ phosphorylation (Figure 9) suggesting its role in p38^MAPK^ in proliferation/survival pathways.

In summary, in the current study we employed the CRISPR/Cas9n technology to target the *Plaur* gene in Neuro 2A neuroblastoma cells. We showed that CRISPR/Cas9n targeting of *Plaur* inhibited cell proliferation. The extent of uPAR downregulation correlated with decrease in full-length TrkC mRNA expression level and downstream intracellular signalling involving Akt and p38^MAPK^.

## MATERIALS AND METHODS

### Guide RNA design and molecular cloning of *Plaur* -specific CRISPR/CAS9n vectors

pX458nickase (D10A) plasmid was generated using pX458 vector (Addgene, # 48138) containing *S. pyogenes Cas9* gene and *EGFP* gene. The point mutation of Asp10 → Ala in the RuvCI endonuclease domain of the *Cas9* gene leads to an improved version of Cas9 – *Cas9n* nickase with enhanced genome editing specificity [43]. For targeting the sequences in the first exon and 5’-UTR of *Plaur*, a pair of gRNAs was chosen using the CRISPR/Cas9n-MIT web tool (http://crispr.mit.edu/) (Figure 1). The oligonucleotide sequences are as follows: sg1 (gRNA1 targeted to 5’-UTR of *Plaur*) 5’CACCGCTGCTCTAGACTGACCGAG 3’; sg1c (complementary to sg1) 5’ AAACCTCGGTCAGTCTAGAGCAGC 3’; sg2 (gRNA2 targeted to the first exon of *Plaur*) 5’CACCGACCCATGGGACTCCCAAGG3’; sg2c (complementary to sg2) 5’ AAACCCTTGGGAGTCCCATGGGTC 3’. The oligonucleotides sg1, sg1c, sg2, sg2c were phosphorylated, denatured and annealed according to the protocol [37]. The obtained 24 bp DNA duplexes were ligated using T4 DNA ligase (Thermo Scientific *#*EL0014) into linearized by BstV2I (SibEnzime *#* E298) pX458nickase vector. Colony PCR method was applied to determine the presence of the insert DNA in the transformed E.coli DH5α [77]. Vector specific primer and primers specific to the minus-strand of the DNA duplex (sg1c or sg2c) (https://www.neb.com/applications/cloning-and-synthetic-biology/dna-analysis/colony-pcr) were used. An amplicon of 700 bp length confirmed the correct orientation. Sequence verification was carried out using seqPrimer 5’ GTTTCGCCACCTCTGACTTGA 3’ (Evrogen).

### Transfection of Neuro 2A cells, flow cytometry and cell sorting

Neuro 2A cells (ATCC^®^ CCL-131™) were maintained in DMEM (PanEko) supplemented with 4.5g/L glucose (Hyclone), 10% FBS (Hyclone), 1% non-essential amino acids (Hyclone) and 100μg/ml penicillin/streptomycin (Gibco^®^) 5% CO_2_ at 37°C. Cells were transfected using Lipofectamine 2000 according to the manufacturer's protocol (Life Technologies). For effective uPAR suppression, we performed three consecutive co-transfection using pX458nickase-sg1 and pX458nickase-sg2 plasmids, followed by cell sorting.

For cell sorting, cells were detached by 0.25% Trypsin/EDTA solution (Gibco^®^), centrifuged at 250g for 5 min and re-suspended with DPBS (Dulbecco's Phosphate-Buffered Saline, PanEko) supplemented with 0.1% BSA. BD FACS Aria III cell sorter equipped with blue laser (*488* nm) and BD FacsDiva software was used to select EGFP-positive cells. EGFP-enriched cell populations after the first, second and third sorting were marked s1, s2 and s3, respectively, and cultured for 2 days.

Cell surface expression of uPAR in s1, s2 and s3 cell populations was analyzed using flow cytometry. Cells were washed with DPBS, detached using Versene solution (PanEko), centrifuged at 250g for 5 min and re-suspended in 100 μl DPBS, containing 0.1% BSA. Cells were incubated sequentially with anti-uPAR antibody (SC-10815, Santa Cruz) and with secondary antibody (AlexaFluor 488, Jackson ImmuneResearch) at 4°C for 30 minutes. Non-specific IgG were used as a negative control. Cells were washed and re-suspended in 500μl DPBS/0.1% BSA.

### Electrophoresis and Western blot analysis

Cells were lysed in RIPA buffer (50 мM HEPES, 100 мM NaCl, 1% sodium deoxycholate, 1% Triton X-100, 0,1% SDS, pH 7.2) containing 4% β-mercaptoethanol and Protease Inhibitor Cocktail (Thermo Scientific). Total protein content was quantified using Bradford assay. After separation by 10% SDS-PAGE electrophoresis, the proteins were transferred to PVDF membrane (GE Healthcare) in transfer buffer (1.92 M Tris/glycine buffer, 10% SDS and 20% methanol). Nonspecific binding was blocked in 5% non-fat dried milk in PBS buffer (Phosphate-Buffered Saline, Sigma), containing 0,1% Tween-20) overnight at 4°C. Proteins were probed with anti-uPAR (Santa Cruz), anti-Akt, anti-p (ser347)-Akt (Cell Signaling #587F11) and anti-p (Thr308)-Akt (Cell Signaling #9275), anti-p38^MAPK^ (Cell Signalling #9212) and anti-phospho-p38^MAPK^ (Abcam #ab32557), β3-tubulin (Santa Cruz #sc-51670), vinculin (Sigmaaldrich V9131), PARP-1 (Santa Cruz sc-7150), caspase 3 (Abcam #ab32351) primary antibodies (1:1000) at room temperature for 2 hours, followed by incubation with secondary antibodies (IMTEK), (1:3000). The proteins were detected using an enhanced chemiluminescence reagent kit (SuperSignal WestPico, Thermo Scientific) and a Chemi-Doc imaging system (Bio-Rad). The proteins were detected using an enhanced chemiluminescence reagent kit (SuperSignal WestPico, Thermo Scientific) and a ChemiDoc™ MP Imaging system (Bio-Rad). Densitometric analysis of blots was performed using ImageJ.

### Quantitative real-time polymerase chain reaction analysis (RT-PCR)

Total RNA from Neuro 2A cells was extracted using an RNeasy^®^ Mini Kit (Qiagen, Germany). To generate cDNA, 1μg of total RNA and MMLV RT kit (Evrogen, Moscow, Russia) was used. PCR was carried out using qPCRmix-HS SYBR (Evrogen, Russia) on a DT-96 real-time PCR device (DNA-technology, Russia). The thermal cycling program for template denaturation, primer annealing and primer extension was 94°C for 15 sec, 62°C for 15 sec and 72°C for 20 sec for 40 cycles, respectively. A relative transcript level of uPAR was calculated using the 2^−ΔΔCt^ method. The following primers for murine uPAR, and β−actin [78], TrkA, TrkB, TrkC, TrkC-FL (full-length) and TrkC-Truncated [65], p75NTR, were obtained from Evrogen (Russia): uPAR-forward 5′-CGCCACAAACCTCTGCAAC-3′, uPAR-reverse 5′-CTCTGTAGGATAGCGGCATTG-3′, β-actin-forward 5′-AGTGTGACGTTGACATCCGTA-3′, β-actin-reverse 5′-GCCAGAGCAGTAATCTCCTTCT-3′, TrkA forward 5’-GCCTAACCATCGTGAAGAGTG-3’, TrkA reverse 5’-CCAACGCATTGGAGGACAGAT-3’, TrkB forward 5’-TGGGACGTTGGGAATTTGGT-3’, TrkB reverse 5’-AGTTGGCGGAAAAAGCACAG-3’, TrkC forward 5’-AGCCACGTCAACCTGACTG-3’, TrkC reverse 5’-CCTCGCTCGTCACGTTCAC-3’, TrkC-FL forward 5’-TGATCCTCGTGGATGGACAG-3’, TrkC-FL reverse 5’-CTTCACTAGTAGATTGGCTCC-3’, TrkC-Truncated forward 5’-CCACTTCCTGAAGGAGCCCT-3’, TrkC-Truncated reverse 5’-CCCACTCTGGACCTCAGGTT-3’, p75NTR forward 5’-ACCCTGCCTGGACAGTGTTA-3’, p75NTR reverse 5’-AGAACACGAGTCCTGAGCCC-3’.

### Neuro 2A cell proliferation analysis *in vitro*

For cell counting analysis, Neuro 2A cells were seeded into 2×12-well at 4×10^4^ cells/well. The cell number was calculated every 24 hours for each cell type and time point using an automatic Countess^®^ Automated Cell Counter (Thermo Fisher Scientific). The proliferation curves were plotted based on the obtained data. Data are presented as the dependence of total cell number on the time interval. The experiment was performed in 2 parallels and repeated three times. Data are presented as the mean ± standard error of the mean, p < 0.05.

Neuro 2A cells were fixed using Fixation&Permeabilization Reagents (eBioscience™) and immunostained with monoclonal antibody (Abcam ab15580) against Ki-67. The percentage of Ki-67-positive Neuro 2A cells in wt culture and uPAR-deficient clones (#6, #30) was analysed using FACS BD FACSCanto™ II Flow Cytometry System. The amount of Ki-67-positive cells, cultured in standard culture media and in low serum conditions (1% FBS), were evaluated after 24, 72 and 96 hours. Data are presented as the mean ± standard deviation, p < 0.05.

### Statistical analysis

Statistical analysis was carried out using statistics program STATISTICA. For pairwise comparisons in case of a normal value distribution, the Student's t-test was implemented; in other cases the Mann-Whitney rank-sum test was used. For multiple comparisons, data were tested for significance using ANOVA (Newman-Keuls test). Differences were considered statistically significant when P < 0.05 was achieved.

## SUPPLEMENTARY MATERIALS FIGURES



## Abbreviations

BSA: Bovine serum albumin
CRISPR: clustered regularly interspaced short palindromic repeats
EGFP: enhanced green fluorescent protein
gRNA: guidance RNA
siRNA: small interfering RNA
uPA: urokinase plasminogen activator
uPAR: uPA receptor
*Plaur*: uPAR gene
5’-UTR: the untranslated 5' terminal region of the mRNA.
