# Supplementary material for: CRISPR/Cas9 nickase mediated targeting of urokinase receptor gene inhibits neuroblastoma cell proliferation
Source: Oncotarget. 2018 Jun 29;9(50):29414–30. doi: 10.18632/oncotarget.25647 (PMC6047682; doi:10.18632/oncotarget.25647)
Supplement: Supplementary file 1 [file oncotarget-09-29414-s001.pdf]

CRISPR/Cas9 nickase mediated targeting of urokinase receptor gene inhibits neuroblastoma cell proliferation

SUPPLEMENTARY MATERIALS

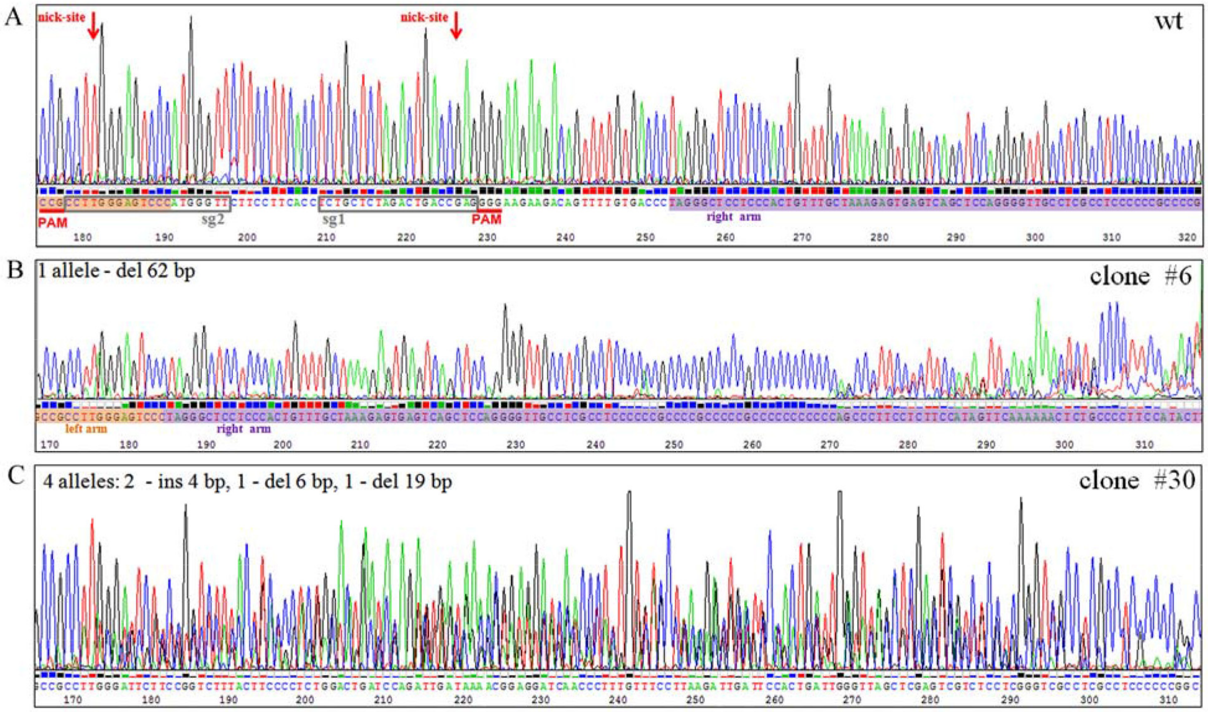

Supplementary Figure 1: Sanger sequencing analysis of *Plaur* sgRNAs on-target sites. Analysis of the sequence data was carried out using ChromasLite and TIDE programs (https://tide.deskgen.com/). **(A)** Sequence of DNA fragments from wild-type (wt) Neuro 2A cells; grey frames depict sgRNAs (sg1 and sg2) target sites, the expected cleavage sites of Cas9n are indicated by red arrows (nick-site), the PAMs are indicated by red line below the nucleotide sequence. **(B)** Sequence of DNA fragments from clone #6 harboring homozygous frameshift (62-nucleotide deletion) resulting in complete uPAR knockout. The gap between the sequences highlighted in orange and violet in wt (A) reflects the resulting 62-nucleotide deletion in clone #6. **(C)** Sequence of DNA fragments from clone #30. Three allele variants have been identified: two alleles bearing identical inserts of 4 nucleotides; one allele with 6 nucleotide deletion potentially restoring the reading frame; and one allele with 19 nucleotide deletion.

A

	Sequence	Score	Mut	Locus
**sg1**	**GCTGCTCTAGACTGACCGAG GGG**	-	-	Chr7:24462471-24462493
**Off1**	**GCTGCTGT-GTCTGACCGAG GGG**	1.79	2 MM, 1 del	Chr16:4839624-4839645
**sg2**	**GACCCATGGGACTCCCAAGG CGG**	-	-	Chr7:24462505-24462527
**Off2**	**GAGCCCTGGGACCCCCAAGG AGG**	1.46	3 MM	Chr19:54959808-54959830
**Off3**	**GACCCATGGGACTACCAA-G CGG**	6.81	1 MM, 1 del	Chr11:88690649-88690670

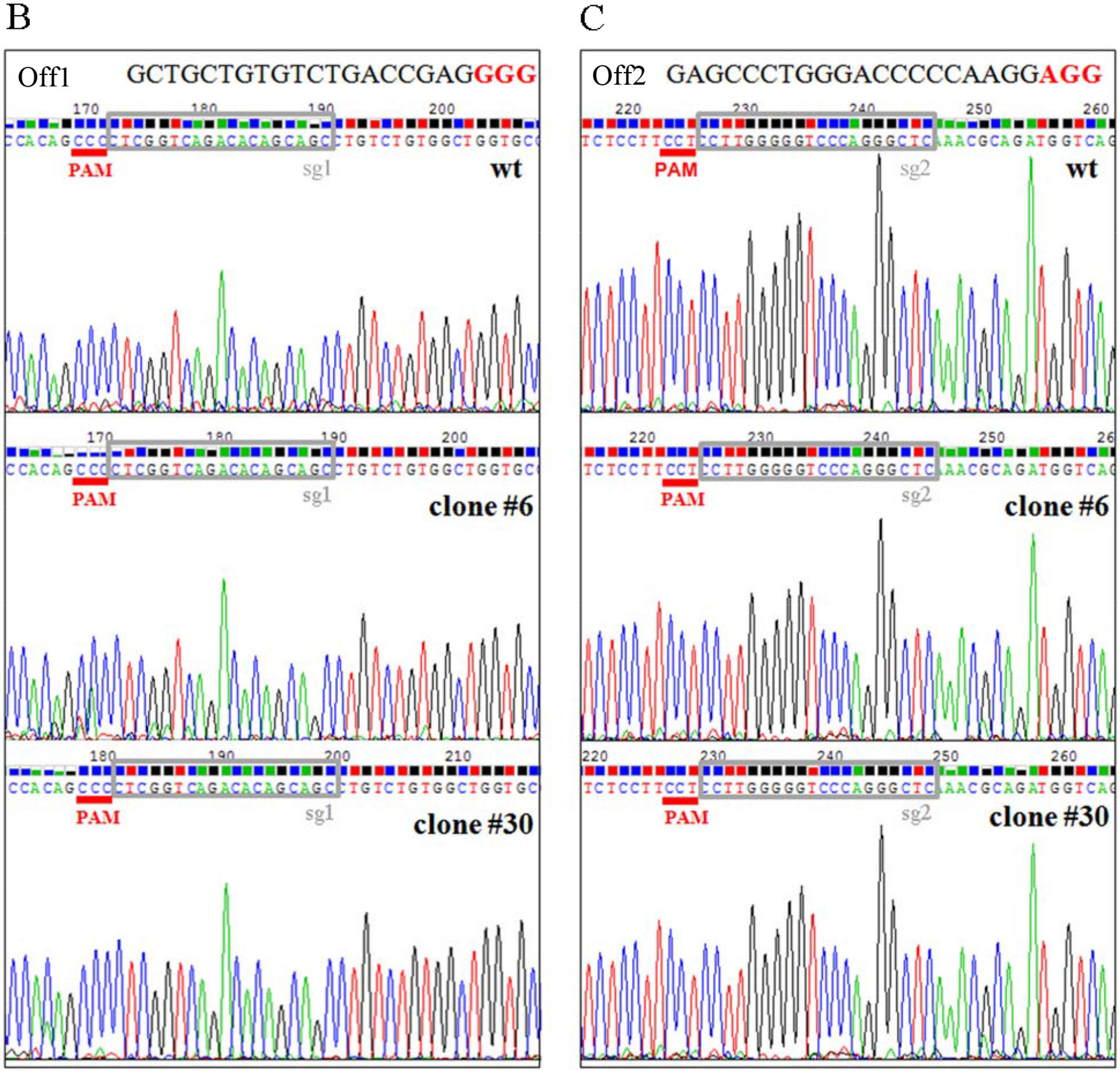

(*Continued*)

D

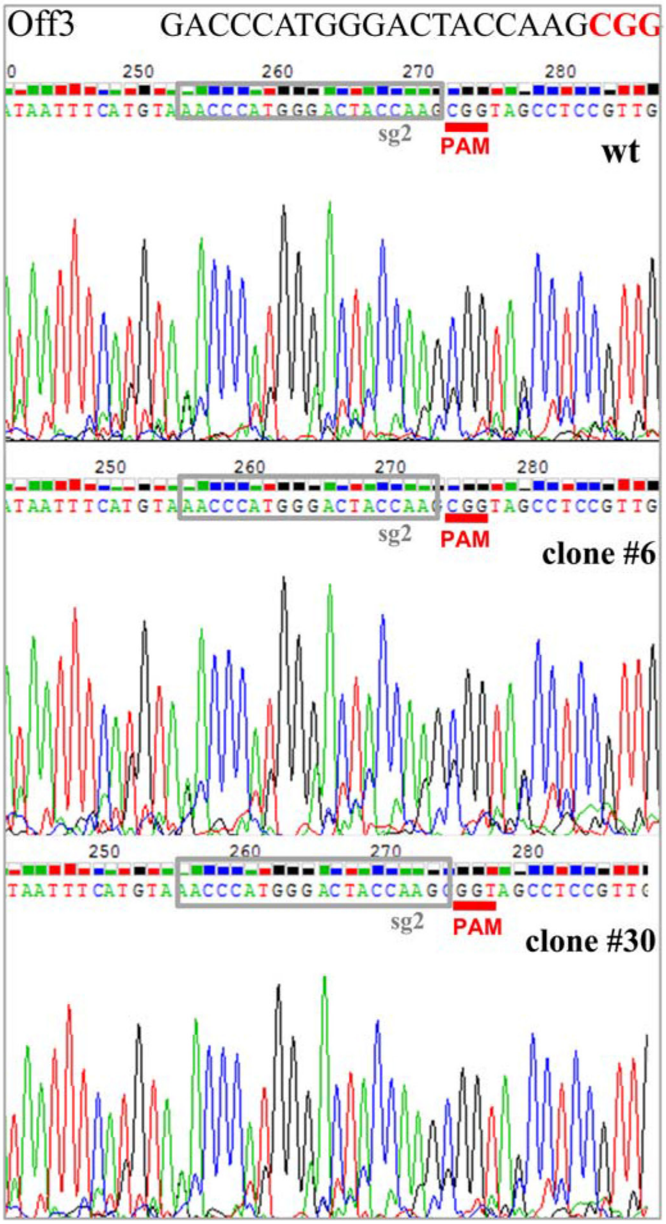

Supplementary Figure 2 (*Continued*): Sanger sequencing analysis of Plaur sgRNAs off-target sites. To predict the most probable Plaur sgRNAs' off-target sites, COSMID software (https://crispr.bme.gatech.edu/) was implemented. **(A)** A list of the top three potential off-target sites, base pair mismatches, and location in the genome. MM-mismatches. **(B-D)** Sequence analysis of DNA fragments from wt cells and clones #6 and #30 at the loci of the most likely off-target activity of the CRISPR/Cas9n. The presence of the intact sequence for each of the clones indicates that there were no mutations in the tested off-target sites. The grey frames depict potential sgRNAs (sg1 and sg2) target sites. The PAMs are indicated by red line below the nucleotide sequence. Analysis of the resulting sequence data was carried out using ChromasLite program.

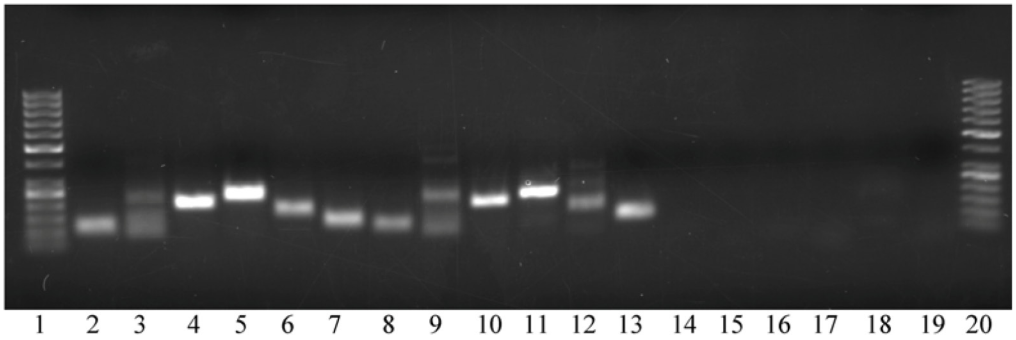

Supplementary Figure 3: RT-PCR analysis of mRNA content from wild-type Neuro 2a cells (wt) and uPAR-deficient clone #6, using primers specific for the TrkA, TrkB, TrkC, p75NTR, uPAR, β-actin gene expression. To verify the production of the single gene-specific product in each sample, we performed agarose gel electrophoresis following RT-PCR. TrkA (99 bp) - lanes 2, 8, 14, TrkB (174 bp) - lanes 3, 9, 15, TrkC (187 bp) – lanes 4, 10, 16, p75NTR (215 bp) – lanes 5, 11, 17, PLAUR (151 bp) – lanes 6, 12, 18, β-actin(112 bp) – lanes 7, 13, 19. 1, 20 - DNA ladder; 2–7 lanes containing primers and DNA template from control Neuro 2A cells; 8–13—probes containing primers and DNA template from uPAR-deficient clone #6 cells; 14–19 - negative control probe containing primers without DNA template.

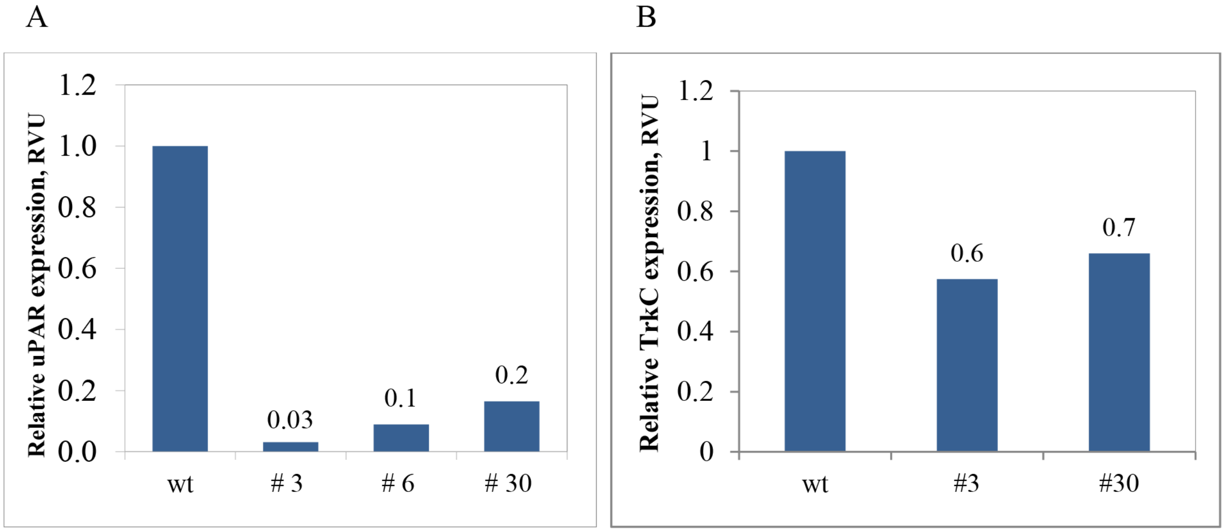

Supplementary Figure 4: RT-PCR analysis on uPAR and TrkC mRNA expression from wild-type (wt) Neuro 2A cells and uPAR-deficient Neuro 2A clones #3, #6, #30. (A) PLAUR mRNA in control cells and in #3, #6, #30 clones; **(B)** TrkC mRNA in control cells and in #3, #6, #30 clones. The mRNA level was normalized to the expression of the housekeeping gene β-actin. Reproducible results of three independent experiments are presented.
